# On Rheumatism

**Published:** 1803-09-01

**Authors:** 


### *To the Editors of the Medical and Phyſical Journal.*

GENTLEMEN,

THROUGH the medium of your excellent publication I learnt the successful result of the practice of Dr. Kinglake in arthritic complaints, by diminished temperature; I have therefore taken the liberty of submitting the following case of rheumatism for your consideration, treated in a similar manner.

F. G. a seaman of one of his Majesty's ships, aged 23, while cruizing on the coast of France in very hazy weather, was attacked in the evening of the 2d of last June, with general acute pains in the limbs and head; great heat and thirst; pulse quick and full. I immediately took sixteen ounces of blood from the arm, and administered the following bolus:

℞. Pulv. antim. (P. L.) gr. iij. calom. gr. v. confect. damoc. q. s. ft. bol. h. som. sumend.

3d. A profuse perspiration in the course of the night relieved, in a tolerable degree, the pains in the upper extremities; but those of the lower ones were augmented, together with one ancle and knee swoln, and considerably inflamed. Head easy, thirst and general indisposition continued, proceeding, I imagined, from the excruciating pain which existed in the lower extremities. I enveloped them in cloths well wet with cold salt water, which, from the excessive temperature reigning, I was obliged to get renewed about every fifteen minutes, as the moisture evaporated as quick as if they had been exposed to a fire. Administered pulv. ipecac. c. h. som.

4th. Upper extremities perfectly easy, and lower ones considerably relieved; inflammation of knee and ancle very much diminished; skin moist, pulse soft, bowels regular, &c. Continued the cold water as before, and repeated the pulv. ipecac. c. at bed-time.

5th. Rested well during the night; phlegmon removed; and tumefaction nearly so. Pains in lower extremities were very trifling; pulse regular, appetite good. I persevered in the use of the same remedies.

6th. Lower extremities perfectly free from pain. Rep. remedia.

7th. In the morning was free from complaint excepting debility; but in the course of the day his wrists became painful, tumefied, and inflamed. I immediately enveloped

*them*

*them* in the same manner I had done the knees and ancles. Rep. pulv. ipecac. c. ut antea.

8th. Pains in the wrists relieved, and the inflammation and swelling almost disappeared. Persevered in the use of the cold water and powder. It is necessary to observe that I continued the cold applications to the knees and ancles, in order to prevent a sympathetic return.

9th. Free from complaints excepting debility and a stiffness in the lower limbs, which I ordered to be rubbed gently with a flesh-brush, and gave him half a drachm of powdered bark mixed with wine four times a day. From this time he began to recover his health and strength, and on the 16th returned to his duty.

I beg leave to observe, that this man about two years ago was attacked in a similar way, and was confined to his bed for six weeks, unable to raise either *hand* or *foot*; and I have every reason to believe, from the violence of *this* attack, that I should have had him confined for the same length of time, if not longer, had I pursued the usual method of cure practised on these occasions; but the sensible and judicious *practice* of Dr. Kinglake in arthritic complaints, pointed out to me a remedy at once effectual, speedy, and *cheap*, for the relief of one of the most painful and tedious complaints incident to the human body; and a complaint that is extremely prevalent in his Majesty's navy, particularly when employed in the channel service.

Before I had an opportunity, owing to an unavoidable absence from my native country, of reading any of your well conducted Journals, I had been in the habit of making use of cold water in local affections of the joints with considerable advantage; I therefore, with a tolerable confidence, hesitated not applying it in the above case.

If you think this single testimony will tend in the smallest degree to corroborate the evidence of your able correspondent in the safety and propriety of using diminished temperature for the relief of painful and inflammatory affections of the joints, whether called by the name of *rheumatism* or *gout*, you are perfectly at liberty to make what use of it you may deem fit; and if *perchance* it should merit your notice, it will amply satisfy your attentive reader.

